# Arterial Ischemic Stroke in Pediatric Patients: A Tertiary Hospital Experience

**DOI:** 10.7759/cureus.72390

**Published:** 2024-10-25

**Authors:** Margarida Camacho-Sampaio, Mariana Costa, Cláudia Lima, Constança Santos, Joana Amaral, Filipe Palavra, Cristina Pereira, Joana Ribeiro

**Affiliations:** 1 Pediatric Neurology, Hospital Pediátrico, Unidade Local de Saúde de Coimbra, Coimbra, PRT; 2 Neurology Department, Instituto Português de Oncologia Francisco Gentil, Coimbra, PRT; 3 Laboratory of Pharmacology and Experimental Therapeutics, Coimbra Institute for Clinical and Biomedical Research (iCBR), Faculty of Medicine, University of Coimbra, Coimbra, PRT; 4 Neurology, Clinical Academic Centre of Coimbra, Coimbra, PRT; 5 Pediatric Neurology, Neurophysiology Department, Reference Centre of Refractory Epilepsies, Hospital Pediátrico, Unidade Local de Saúde de Coimbra, Coimbra, PRT; 6 Neurology, Faculty of Medicine, University of Coimbra, Coimbra, PRT

**Keywords:** arterial ischemic stroke, childhood, ischemic stroke, pediatrics, pediatric stroke

## Abstract

Introduction: Childhood arterial ischemic stroke is a rare condition. Its true incidence is unknown as it is likely underdiagnosed. Outcomes are generally favorable but it is still a significant cause of mortality and morbidity in children. We aimed to characterize patients with childhood arterial ischemic stroke regarding risk factors, etiology, treatment, and outcome.

Methods: This was a retrospective observational study that analyzed demographic and clinical data of patients aged 28 days to 18 years of age diagnosed with arterial ischemic stroke and admitted to the neuropediatric department at a tertiary hospital in Coimbra, Portugal, between 2015 and 2022.

Results: A total of 19 patients were included, with a median age of 12.13 years; 10 (53%) were male children and 13 (63%) had a pre-existing medical condition. Eleven patients (58%) presented with sudden focal deficit and eight (42%) with only unspecific/non-localizing symptoms (headache, altered mental status, and seizure). The middle cerebral artery was the most affected (n=13, 68%) and regarding etiology, seven (37%) were cardioembolic and another seven (37%) were unilateral focal cerebral arteriopathy. Conservative measures were used for the treatment of 11 (90%) patients and two underwent mechanical thrombectomy successfully. Regarding sequelae, four patients (21%) had moderate to severe disability. The main sequelae was chronic motor deficit (n=8, 42%), followed by cortical deficit (n=4, 21%). Two patients developed vascular epilepsy.

Conclusions: While most patients presented with a sudden focal deficit, the occurrence of non-localizing symptoms was high. Conservative treatment was the most used, but there were good results in patients submitted to invasive treatment. Our results corroborate the high morbidity associated with childhood arterial ischemic stroke and the need for a high clinical suspicion to identify these cases and treat them promptly.

## Introduction

In developed countries, cerebrovascular diseases are among the 10 most common causes of death [1,2]. Stroke is traditionally classified into ischemic and hemorrhagic subtypes. This article focuses on childhood arterial ischemic stroke (AIS). Childhood AIS is the acute onset of neurological signs or symptoms attributable to brain infarction and occurs between the ages of 28 days and 18 years [3,4].

Childhood AIS differs considerably from AIS in adults, being rarer and with various manifestations, including unspecific symptoms often resulting in delayed or missed diagnoses [1,5]. Stroke mimics in children are frequent and include Todd paresis, migrainous aura, and functional neurological symptoms [3,6]. In around 79-93% of pediatric patients with acute focal brain dysfunction, the underlying cause will be a stroke mimic [5]. There is a lack of comprehensive data on outcomes in children, such as stroke volume, recurrent strokes, and long-term disabilities [1].

Childhood AIS is a relatively rare condition, with an estimated incidence of three cases per 100,000 each year [1]. However, its true incidence is unknown, as it is likely underdiagnosed and underreported [7]. Incidence is higher in children under five years of age, with a slight male prevalence. Black children are at higher risk for AIS, due to the higher prevalence of sickle cell disease (SCD) in this group [3,4].

The most important and frequently identified cause of childhood AIS is cerebral arteriopathy, responsible for 21-53% of AIS in children, and presenting mostly as focal cerebral arteriopathy of childhood (FCA) [8,9]. FCA typically occurs in the context of inflammation or infection, and its association with varicella-virus infection is well established [6,10,11]. Another relevant type of cerebral arteriopathy in pediatric patients is Moyamoya disease, a progressive stenotic disease of the anterior circulation intracranial vessels. It can be idiopathic or, more commonly, associated with (i) trisomy 21, (ii) neurofibromatosis type 1 (NF-1), (iii) SCD, (iv) prior radiation therapy to the head and neck, (v) arterial structural or inflammatory vasculopathy (in the setting of connective tissue disorders, fibromuscular dysplasia, and Takayasu disease) [6,10,11].

The next two most common causes of childhood AIS are cardioembolism (24-31%), mainly in the setting of congenital or acquired heart disease, and artery dissection (20%), responsible for up to 50% of strokes in the posterior circulation, typically in the setting of head or neck trauma [3,6,9]. Other important causes include SCD, associated with a higher risk of cerebral ischemia in children when compared with adults [2]. It causes stroke due to hyperviscosity and SCD-associated arteriopathy [2,3]. Hypercoagulable states, either genetic or secondary to chronic systemic illness, are also relevant in AIS, especially when in combination or in the presence of patent foramen ovale (PFO). Prothrombotic mutations can be found in up to 13% of children with ischemic stroke [2,11]. Many pediatric AIS are multifactorial rather than related to a single etiology, with overlapping genetic predisposition and acquired risk factors [6,11]. Of childhood strokes, 10-25% remain cryptogenic [2,10].

While most children with AIS present with a sudden focal deficit (hemiparesis with facial weakness in 67-90%, speech or language disturbance in 20-50%, vision disturbance in 10-15%, and ataxia in 8-10%), the occurrence of non-localizing symptoms such as headache or loss of consciousness is very high (20-50% and 17-38%, respectively). Children may have stepwise rather than abrupt symptom onset. About 25% have symptomatic seizures at presentation, and this percentage is higher in children under one year of age, unlike headache, which occurs more frequently in children older than five [3,6]. Posterior circulation strokes are particularly challenging since their common presentation includes headache and emesis, which are also common symptoms in other childhood diseases. Additionally, children may have a difficult time describing vertigo or dizziness, but these strokes are usually (70-100%) accompanied by deficits referable to the posterior circulation such as hemiparesis, ataxia, dysarthria, visual field deficits, and oculomotor deficits [3]. Around a third of children with AIS have a previous history of transient ischemic attacks (TIA), which are particularly common with arteriopathy, and are the norm in Moyamoya syndrome [12]. The median time from symptom onset to parent seeking medical care is highly variable, ranging from 1.7 to 21 hours, although a majority reach medical care within six hours [3].

Magnetic resonance imaging (MRI), particularly with the use of diffusion-weighted imaging (DWI) in the hyperacute period is the preferred imaging modality for acute stroke in children with reported sensitivity ranges from 88-100% vs 60% in computerized tomography (CT) in early AIS. Neurovascular evaluation should be done urgently; magnetic resonance angiography (MRA) of the neck and head (three-dimensional time-of-flight) without gadolinium is useful to evaluate the extracranial and intracranial large arteries and to select patients for acute therapies. However, CT is used more often because of its widespread availability, rapid scan times, and lower cost. It has the added benefit of allowing for immediate diagnosis of occlusive arteriopathy, which facilitates the choice of acute-phase therapy [11].

Acute treatment of childhood AIS focuses on neuroprotective management [2]. Intravenous (IV) tissue plasminogen activator (tPA) and revascularization, which are revolutionary in managing adult AIS, are still lacking evidence supporting their use in children [7]. In older children with imaging confirmation of vessel occlusion and stroke, it may be reasonable to consider IV thrombolysis within the first 4.5 hours and mechanical thrombectomy (MT) within the first six hours of symptom onset [2]. Mounting observational data suggests that MT for acute stroke has a good safety profile in children [12]. Despite the advances in treating AIS in children, the hallmarks of acute and chronic therapies include anticoagulation and antiplatelet medications [2]. The 2008 American Heart Association scientific statement generally support either initial aspirin or low-molecular-weight heparin (LMWH) for initial therapy in pediatric AIS. In most children, continued maintenance therapy consists of aspirin for two years (to cover the time window when the vast majority of recurrent strokes occur) [3].

Stroke is nonetheless a significant cause of mortality and morbidity in children. Mortality rate ranges between 2.6% and 5% [1,9]. Two years after stroke, most children will have good neurological function [10]. However, a significant minority will have persistent neurologic deficits, ranging from focal neurologic signs to learning disability, cognitive impairment, behavioral problems, and vascular epilepsy [10,13]. The prevalence of long-term cognitive and behavioral problems is difficult to ascertain, reflecting the variable practices in follow-up and clinical assessment between centers [13]. Approximately 20% will have stroke recurrence, with the risk being highest within the first year of the first stroke [9]. This risk is higher in children with complex cardiac disease, persistent arteriopathy, SCD, or significant thrombophilia [6].

## Materials and methods

This was a retrospective observational study conducted at the Pediatric Neurology Department in Hospital Pediátrico, Unidade Local de Saúde de Coimbra, Portugal. The study was approved by the Ethics Committee of Centro Hospitalar e Universitário de Coimbra (approval number: OBS_SF_057_2023). Due to the retrospective nature of the study, informed consent was waived by the ethics committee.

All patients with childhood AIS assisted in the Pediatric Neurology Department between 2015 and 2022 were considered. The inclusion criteria were patients aged 28 days to 18 years with a diagnosis of AIS confirmed by CT or MRI reviewed by experienced neuroradiologists. Patients with perinatal stroke, aged less than 28 days, and with hemorrhagic stroke were excluded.

Data were collected from clinical records. Variables included in the study were demographic data, concomitant diseases, clinical syndrome (Oxfordshire classification), classification of severity using the Pediatric National Institutes of Health Stroke Scale (PedNIHSS), and outcome ascertained using the Modified Pediatric Ranking Scale (mRS), a clinician-reported measure of global disability varying between 0-7 (no symptoms to death) [14-16].

The etiology of AIS was classified using the Childhood AIS Standardized Classification and Diagnostic Evaluation (CASCADE) criteria, which divides pediatric AIS causes into seven categories: small vessel arteriopathy; unilateral FCA (further divided as FCA-inflammatory and FCA-dissection), bilateral cerebral arteriopathy, aortic/cervical arteriopathy, cardio-embolism, other (encompassing undetermined cause after complete investigation), and multifactorial [2,17-18]. 

The medical records of patients were reviewed and then fed into a standardized Excel extraction form (Microsoft Corporation, Redmond, Washington, United States). Data descriptive analysis was performed with IBM SPSS Statistics for Windows, Version 29.0 (Released 2023; IBM Corp., Armonk, New York, United States). Categorical variables are presented as frequencies and percentages, and continuous variables as means and standard deviations (SD) if normally dis­tributed. Normal distribution was verified through the Kolmogorov-Smirnov test.

## Results

**Figure 1:**
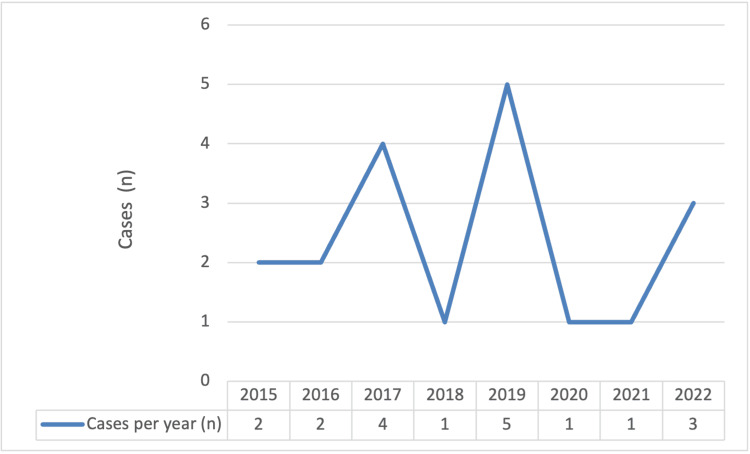
Cases per year

A total of 19 patients with pediatric AIS who were assisted in our department between 2015 and 2022 were included in the study. Of these, 53% (n=10) were boys. The number of cases per year was highly variable, with a mean of 2.38 (SD 1.5) per year (Figure 1). Our department serves a population of around 300,000 children, with a median of 60,103 admissions to the emergency department per year, resulting in an approximate annual incidence of childhood AIS of 0-2 (mean 0.8) per 100,000 (data from the National Institute of Statistics Portugal, 2021) [19]. It bears mentioning that of the sample, three (16%) were Black children and from the community of Portuguese-speaking countries (CPLP). 

Median age at diagnosis was 12.13 years (interquartile range (IQR) 3.9-15.9 years). Table 1 shows the demographic characteristics of the patients.

**Table 1:** Demographic characteristics of patients (N=19)

Characteristics	Frequency (Percentage)
Gender	
Male	10 (53%)
Age in years	
<2	4 (21%)
2-13	7 (37%)
13-18	8 (42%)

Only six (32%) patients were previously healthy, and 13 (68%) had a pre-existent medical condition: cardiac heart disease (congenital heart disease (n=5) and rheumatic valvopathy (n=2)), familiar hypercholesterolemia (n=1), SCD (n=1), NF-1 (n=1), medulloblastoma (n=1), hemolytic uremic syndrome (n=1), chromossomopathy (duplication of the Xq27.3-q28 region) (n=1), and migraine with aura (n=2). Two patients had more than one pre-existing medical condition.

The symptoms and signs at presentation are represented in Table 2 and Table 3. Eleven patients (58%) presented with one or more sudden focal deficits: hemiparesis with/or facial weakness (n=8, 42.1%), speech or language disturbance (n=4, 21.1%), ataxia (n=3, 15.8%), and vision disturbance (n=1, 5.3%). Eight (42%) patients had only unspecific/non-localizing symptoms (headache, altered mental status, and seizure). Although the majority had localizing symptoms, three presented with seizures and four with intense headaches before the appearance of focal deficits. Two (11%) patients had a history of previous TIA. The PedNHISS at admission was determined in 15 (79%) patients and ranged between 1 and 18, with a median of 4. Childhood AIS was diagnosed with a median delay of 23 hours post symptom onset.

**Table 2:** Patient characteristics, etiology, and symptoms MCA: middle cerebral artery; NA: not available; NF-1 neurofibromatosis type 1; PACS: partial anterior circulation stroke; PCA: posterior cerebral artery; PedNHISS: pediatric National Institutes of Health Stroke Scale Score; PICA: posterior inferior cerebellar artery; PFO: patent foramen ovale; POCI: posterior circulation infarcts; SCD: sickle cell disease; TACS: total anterior circulation stroke

Etiology		Age	Gender	Oxfordshire classification	Vascular territory	Ped NHISS	Symptoms	Pre-existing medical condition
Unilateral focal cerebral arteriopathy	Moya Moya disease	11 years	M	Left LACS	Left MCA	NA	Headache, right hemiparesis, and vomiting	Cromossomopathy, Interatrial communication
		8 months	F	PACS right	Right MCA	3	Seizure, left hemiparesis	NF-1
	Varicella virus	4 years	M	POCI	Right PICA	4	Ataxia, vomiting and headache	Healthy
		2 years	M	PACS right	Right MCA	3	Ataxia, seizure	Healthy
	Tuberculous meningitis	16 years	M	Other	Right MCA	1	Headache, vomiting, altered mental status, dysarthria	Healthy
	Unknown microorganism	7 years	F	PACS left	Left MCA	4	Headache, seizure	Healthy
	Radiation arteriopathy	17 years	F	POCI	Vertebral artery	NA	Pale, diaphoretic, cyanotic, headache and altered mental status	Medulloblastoma
Cervical arteriopathy	Extrinsic compression of the internal carotid	16 years	M	PACS left	Left MCA	3	Paresthesis and dysarthria	Healthy
Cardioembolic	Intracavitary thrombus	8 years	F	TACS right	Right MCA	10	Seizure	Hemolytic uremic syndrome
	Iatrogenic - valvuloplasty	15 years	F	TACS right	Right MCA	10	Left hemiparesis	SCD, valvulopathy
		15 years	F	PACS right	Right MCA	7	Left hemiparesis and altered mental status	Valvulopathy
		2 months	M	Other	Multiple	NA	Seizure	Transposition of the great vessels
	Congenital heart disease	14 years	M	POCI	Right PICA	1	Headache vertigo, vomiting	PFO major
		5 months	F	Other	Right MCA	NA	Seizure	Fallot's tetralogy
		1 year	M	TACS right	Right MCA	12	Irritability, left hemiparesis, lip corner deviation to the right	Fallot's tetralogy
Indeterminate	Indeterminate	12 years	F	TACS left	Left MCA	18	Right hemiparesis	Familial Hypercholesterolemia; Migraine
		12 years	M	POCI	Basilar artery	11	Headache, vomiting. Seizure. Dysarthria. Left hemiparesis	Hemolytic uremic syndrome
		16 years	F	PACS left	Left MCA	8	Ieritability, disartria, left facial weakness	PFO, Migraine
		17 years	M	POCI left	Left PCA	1	right fronto-parietal headache, contralateral hemianopsia	Healthy

**Table 3:** Symptoms and signs at presentation ^*^Patients that presented with unspecific symptoms before the appearance of focal deficits. Data given as frequency (and frequency (percentage) where specified).

Symptoms and signs	Frequency
Only unspecific/non-localizing symptoms (headache, altered mental and/or seizure), n (%)	8 (42%)
Sudden focal deficit, n (%)	11 (58%)
- Hemiparesis with/or facial weakness	8
- Speech or language disturbance	4
- Ataxia	3
- Vision disturbance	1
- Seizure before focal deficits*	3
- Headache before focal deficits*	4

**Figure 2:**
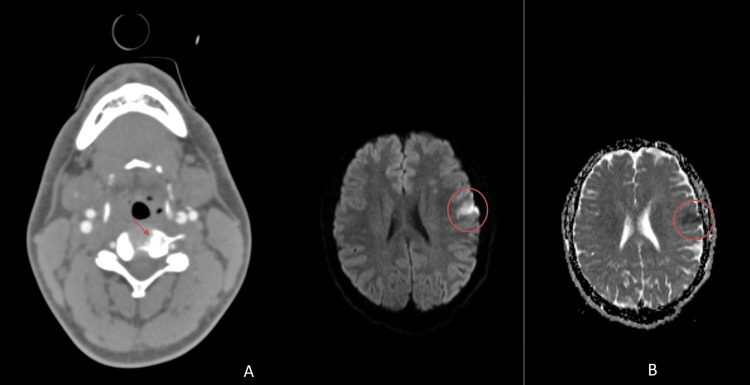
⁠(A) CTA shows a subtle indentation on the medial wall of the initial segment of the left internal carotid artery due to proximity to the left greater horn of the hyoid bone; (B) MRI, DWI shows restricted diffusion in the left posterior frontal area CTA: computerized tomography angiography; DWI: diffusion-weighted imaging; MRI: magnetic resonance imaging

**Figure 3:**
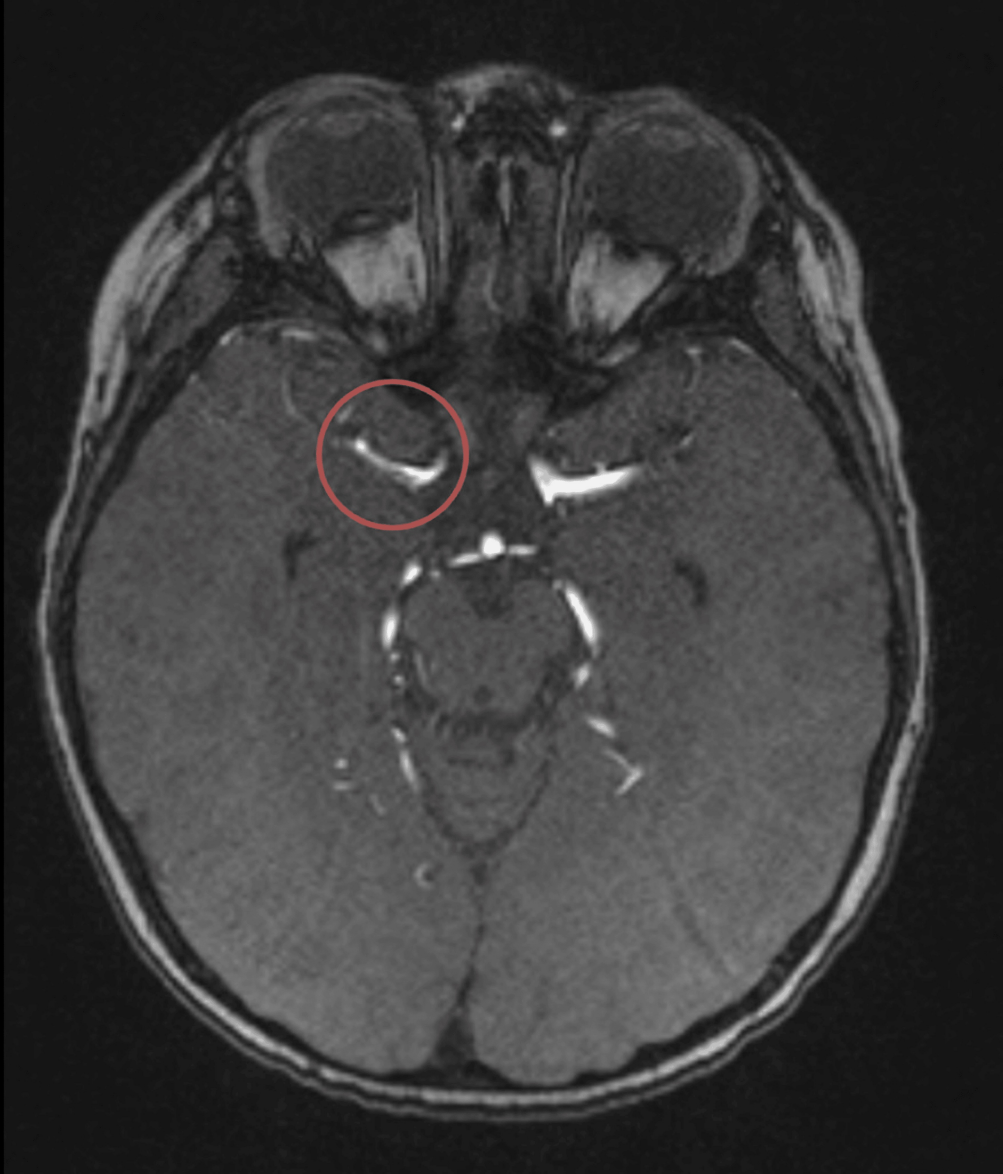
Magnetic resonance angiography TOF shows a subocclusive stenosis of the initial segment of the MCA (FCA-I due to varicella virus infection) FCA-I: focal cerebral arteriopathy of childhood inflammation type; MCA: middle cerebral artery; TOF: time of flight angiography

The CASCADE classification revealed the following causes: cardioembolic (n=7, 36.8%), unilateral FCA (n=7, 36.8%), cervical arteriopathy (n=1, 5.4%) (Figure 2), and other (n=4, 21.1%). In the unilateral FCA group, we had two (10.5%) patients with Moyamoya disease (one also having NF-1), four (21.1%) with FCA-I (two associated with varicella-virus infection (Figure 3), one with tuberculous meningitis and one with presumed infection of unknown cause), and one (5.4%) with radiation arteriopathy (history of medulloblastoma). In the cardio-embolic group, four (21.1%) patients had congenital heart disease (two with Fallot tetralogy, one with transposition of the great vessels, and one with major PFO), two (10.5%) had rheumatic valvulopathy and one (5.4%) had an intracavitary thrombus in the context of hemolytic uremic syndrome. In three (15.8%) patients, the AIS occurred after a cardiac surgical procedure (in the first 24 hours after surgery in two cases; and 12 days after surgery in another).

**Figure 4:**
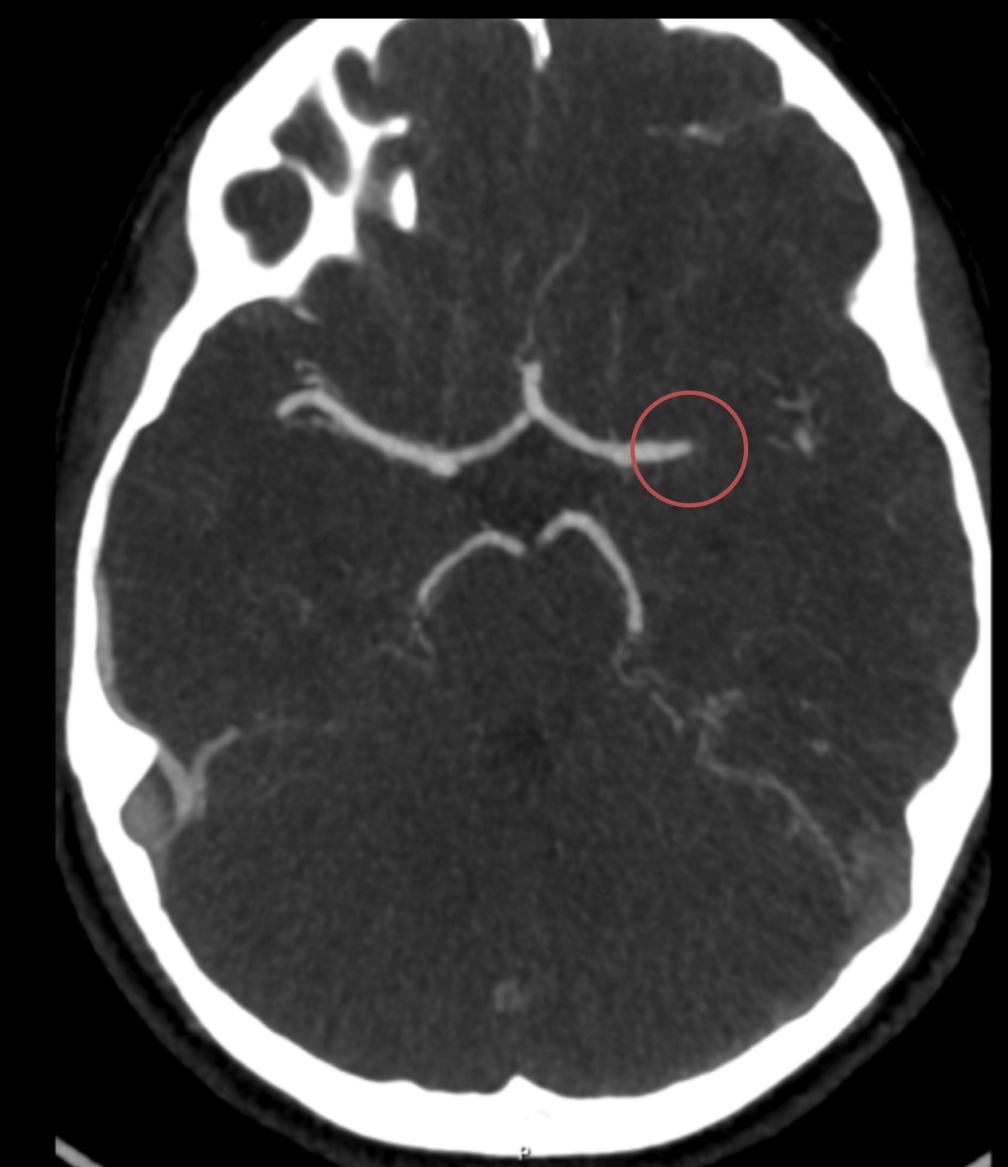
CTA shows occlusion of the first segment of the MCA CTA: computerized tomography angiography; MCA: middle cerebral artery.

The majority (n=17, 89.5%) was treated only with conservative measures, including antiplatelet therapy in all, except two patients who were anticoagulated. Two patients were submitted to a successful MT in 2022, with a PedNHISS of 18 and 12 at admission, and 1 and 11 at follow-up. The first case involved a 23-month-old male patient with Tetralogy of Fallot and right-sided total anterior circulation stroke (TACS) that affected the territory of the right middle cerebral artery (MCA). The second case involved a 12-year-old girl with a history of familial hypercholesterolemia and migraine with aura medicated with statins, that presented with a left MCA occlusion (Figure 4). The time since symptom onset and recanalization was 308 and 345 minutes, respectively. The decision to proceed with MT was made after discussion with the child neurologist and the vascular stroke team. No patients were submitted to tPA. None had hemorrhagic transformation. There were no deaths or AIS recurrence in our patients.

Patient follow-up (n=18) is represented in Table 4. mRS score at follow-up ranged between 1 and 5. In four (17%) patients, mRS score was greater than 3 (moderate to severe disability). All patients maintained follow-up until they reached adulthood or until transfer to another hospital due to a change in residence. Follow-up was lost in one (5.8%) patient due to returning to his country of origin. The main sequelae observed were chronic motor deficit, present in eight (42%) patients, followed by cortical deficit (aphasia/neglect) in four patients (21%). Two (11%) patients had visual deficits, and one patient had ataxia. Two (11%) patients developed vascular epilepsy. Four (21%) children required a special/adapted school curriculum due to learning difficulties.

**Table 4:** Sequelae of patients at follow-up NA: not available; mRS: modified pediatric Ranking Scale; PedNHISS: Pediatric National Institutes of Health Stroke Scale Score

Etiology		Age	PedNHISS	mRs follow-up	PedNHISS follow-up	Motor deficit	Cortical deficit	Sensory deficit	Ataxia/cerebellar signs
Unilateral focal cerebral arteriopathy	Moya Moya disease	11 years	NA	1	2	No	No	No	No
		8 months	3	3	2	No	No	No	No
	Varicella-virus	4 years	4	1	2	No	No	No	No
		2 years	3	1	0	No	No	No	No
	Tuberculous meningitis	16 years	1	0	0	No	No	No	No
	Indertemined germen	7 years	4	2	1	Yes	No	No	No
	Radic arteriopathy	17 years	NA	5	NA	Yes	Yes	Yes	Yes
Cervical arteriopathy	Extrinsic compression of the internal carotid	16 years	3	1	1	Yes	No	No	No
Cardioembolic	Intracavitary thrombus	8 years	10	0	0	No	No	No	No
	Iatrogenic - valvuloplasty	15 years	10	1	2	No	No	No	No
		15 years	7	1	NA	NA	NA	NA	NA
		2 months	NA	NA	NA	NA	NA	NA	NA
	Congenital heart disease	14 years	1	0	0	No	No	No	No
		5 months	NA	0	0	No	No	No	No
		1 year	12	4	11	Yes	Yes	No	No
Indeterminate	Indeterminate	12 years	18	0	0	No	No	No	No
		12 years	11	4	1	Yes	Yes	No	No
		16 years	8	1	1	Yes	Yes	No	No
		17 years	1	1	2	No	No	No	No

## Discussion

The incidence of AIS in our population was lower than described in the literature (1:100,000 vs 3:100,000 cases per year) [3]. This highlights the need for increased awareness of AIS which can remain underdiagnosed without adequate medical recognition. It is known that Black children are at higher risk of AIS, due to their higher prevalence of SCD [1,2]. Our sample only had one child with SCD, which can help to explain our lower incidence rate. Furthermore, in the context of SCD, there are specific protocols primarily oriented toward the systematic monitoring of affected individuals to mitigate stroke incidence, which can also explain the low prevalence of SCD-associated AIS [1,10]. 

According to the literature, AIS is more common in children under the age of five, with a slight male predominance. In our sample, although a slight male predominance was observed, only 32% of the children were under the age of five [3]. Similarly to other studies [14,21], only a minority of patients (32%) were previously healthy. This highlights the importance that widespread awareness of risk factors, especially cardiac heart disease, can have in early diagnosis and treatment.

Regarding the CASCADE classification, cardioembolism and unilateral FCA were the main causes of childhood AIS, with the same number of cases. These results are similar to those described in the literature [1,8,9]. Congenital or acquired heart disease were the main cause of cardioembolism, and in three cases, they were associated with surgical procedures, which should be clinically evaluated in the surgical setting. One adolescent patient had a major PFO that was assumed to be the cause of paradoxical embolization and subsequent AIS. According to the literature, this condition does not have a well-established causal effect with AIS, as it is very common in the general population, and the timing of physiological closure varies significantly [3,4,11]. FCA was also a significant etiology and FCA-I was identified in four patients, two of them with varicella virus infection, a condition with an established association with childhood AIS [6,10,11]. In Portugal, varicella virus vaccination is not included in the national vaccination program, and the incidence of vasculopathy related to this infection may become important to plan for healthcare resources. Moyamoya disease is also a significant cause of AIS and can be idiopathic (as in one of our patients) or associated with other conditions such as NF-1 (as in another of our patients). Of the two patients with a history of TIA, one had Moyamoya disease [15,22].

Although more than half (58%) presented with a sudden focal deficit, a considerable number of patients (42%) presented with non-localizing and very unspecific symptoms, which makes the diagnosis of AIS harder and requires a higher level of suspicion. Even in some patients with focal deficits, the initial manifestations were nonspecific signs and symptoms. These findings are consistent with those reported in the literature [3,6]. This further illustrates the need for raising awareness among caregivers about pediatric AIS and its presentation, especially in patients with known risk factors or with recent varicella-virus infection. 

In our population, none of the patients was submitted to tPA, which can be explained by diagnostic delay. AIS was diagnosed with a median delay of 23 hours post symptom onset, which can be attributed to several factors: a lack of awareness of childhood AIS among caregivers and healthcare professionals, barriers to accessing timely neuroimaging, and a high frequency of stroke mimics in children [16]. The Thrombolysis in Pediatric Stroke Trial offered potential criteria to safely utilize tPA in children, but these criteria have not been widely accepted. Currently, the use of IV thrombolytic agents is increasing. In the United States, it is currently administered in approximately 5-7% of cases of childhood strokes [11]. Despite the increased use of thrombolytics for acute childhood stroke, the true benefit conferred by tPA, the ideal weight-based dosing, and the optimal time window for administration in this population remain unknown [11]. In our sample, the timing of AIS diagnosis and the risk of hemorrhagic transformation with thrombolysis in patients with consolidated infarct precluded the utilization of tPA.

The two cases that underwent MT had good outcomes (mRS of 0 and 4; PedNIHSS after stroke of 0 and 1, respectively). These results are similar to those described in the Save ChildS study, which showed that patients had favorable neurologic outcomes six months after stroke, with 87% achieving an mRS of 0-2. There are other centers with the same experience in Portugal [20]. The time window to safely perform MT is generally extrapolated from adult data, but differences in collateral vasculature or other pediatric-specific factors may modify the time window in children [20,21]. As there are no randomized controlled data that allow for clear recommendations about when to proceed with thrombectomy in children, a multidisciplinary and case-based decision was paramount in our patients.

Regarding sequelae, 25% had moderate to severe disability (mRS score > 3), 40% had a chronic motor deficit, and 21% had a cortical deficit. Only two patients (11%) developed vascular epilepsy, which differs from the approximately 30% reported in the literature [6]. In one of the cases, AIS manifested with seizures. For both cases, the etiology was FCA, one with Moyamoya disease and the other associated with varicella virus.

**Figure 5:**
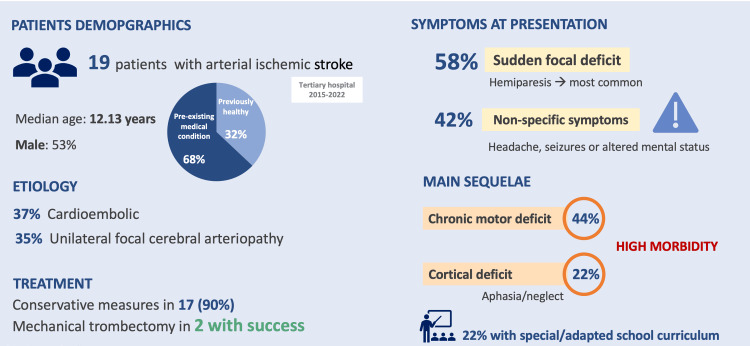
Major points of the study

The major findings of the study are summarized in Figure 5.

Some of the limitations of this study include the retrospective nature of the data collected and the small sample size, which can be explained by the rarity of the disease. Conversely, the minimal follow-up loss and the fact that it is one of the few studies on ischemic stroke in a pediatric setting in Portugal can be valuable contributions to a better understanding of this disease. Future multicentric studies and/or international registries will be key to spreading information regarding risk factors, presentation, and management of such patients.

## Conclusions

In our study, while most patients presented with a sudden focal deficit, the occurrence of non-localizing symptoms such as headache or loss of consciousness was high. Our results corroborate the high morbidity associated with childhood AIS and the need for a high clinical suspicion. Although conservative treatment is the most common approach, we obtained good results in the two patients who underwent invasive treatment with MT. It is crucial to further investigate AIS in childhood in order to collect reliable data on the presentation, risk factors, etiology, and treatment of this condition, and therefore improve diagnosis and treatment in order to prevent the associated morbidity.
